# Cardiovagal Tone in Generalised Anxiety Disorder: A Case Control Study

**DOI:** 10.7759/cureus.103595

**Published:** 2026-02-14

**Authors:** Anu Priya, Sarvesh Malaki, Pallavi Abhilasha, Bharat Singh, Sandeep Singh, Ashly P Koshy, Jeyaraj D Pandian

**Affiliations:** 1 Department of Psychiatry, Christian Medical College, Ludhiana, IND; 2 Department of Neurophysiology, Christian Medical College, Ludhiana, IND; 3 Department of Community Medicine, Christian Medical College, Ludhiana, IND; 4 Department of Neurology, Christian Medical College, Ludhiana, IND

**Keywords:** autonomic dysfunction, cardiovagal tone, cardiovascular risk, ewing’s battery, generalised anxiety disorder, heart rate variability, parasympathetic regulation

## Abstract

Introduction

Generalised anxiety disorder (GAD) is a prevalent psychiatric condition increasingly associated with autonomic nervous system dysfunction. Specifically, impaired cardiovagal tone, a marker of reduced parasympathetic modulation of cardiac function, has been linked to emotional dysregulation, behavioural rigidity, and elevated cardiovascular morbidity. The standard Ewing’s battery of parasympathetic autonomic function tests provides a reproducible and non-invasive means of assessing such dysfunction. This study aimed to comprehensively evaluate cardiovagal tone in newly diagnosed, treatment-naive GAD patients using validated Ewing’s tests.

Methods

In this cross-sectional case-control study, 40 GAD patients (aged 31 to 40 years) diagnosed per the Diagnostic and Statistical Manual of Mental Disorders (DSM)-5 criteria were compared with 40 age- and sex-matched healthy controls. Cardiovagal tone was assessed using the standard Ewing’s battery: heart rate response to deep breathing (HRDB), expiration:inspiration (E:I) ratio, Valsalva ratio, and the 30:15 ratio during the head-up tilt test (HUT).

Results

The GAD patients demonstrated significantly lower HRDB (21.61 ± 9.75 vs. 27.35 ± 9.64 beats/min, p = 0.009) and E:I ratio (1.29 ± 0.26 vs. 1.47 ± 0.20, p = 0.001) compared to controls, indicating reduced parasympathetic (vagal) activity. The Valsalva ratio (p = 0.410) and 30:15 ratio (p = 0.664) showed no statistically significant differences. These findings underscore the presence of early, subclinical autonomic imbalance in GAD, particularly affecting the cardiovagal domain.

Conclusion

Treatment-naive GAD patients exhibit significant cardiovagal dysfunction, as reflected by reduced heart rate variability and E:I ratio. Impaired cardiovagal tone is an independent risk factor for adverse cardiovascular outcomes and may serve as a physiological marker of early brain-heart axis disruption. Routine application of Ewing’s parasympathetic test battery may assist in cardiovascular risk stratification and guide holistic neurocardiac management in anxiety disorders.

## Introduction

The autonomic nervous system (ANS), comprising the sympathetic and parasympathetic systems, influences multiple involuntary reflexes, ensuring precise regulation of physiological parameters and modulating responses to the body's needs. A healthy ANS reflects an individual's ability to adapt to their surroundings, offering physiological flexibility that enables the body to respond to various environmental and internal challenges, thereby promoting overall health and resilience. A disruption in the tight regulation of essential variables, including heart rate (HR), blood pressure, respiratory rate, and pattern, can have long-term consequences for an individual's overall health and quality of life, predisposing them to chronic cardiovascular, gastrointestinal, and psychological disorders [1].

The parasympathetic influence on the heart, exhibited by the cardiovagal tone, exerts a protective effect. Conversely, decreased cardiovagal tone is an established risk factor for overall cardiovascular health, including an increased risk of coronary heart disease, progression of atherosclerosis, and major adverse cardiac events [2,3]. It also plays an important role in cognitive, social, and emotional behaviour and is influenced by the emotional and psychological states of an individual, establishing a crosstalk between the brain and the heart, wherein psychological stresses, including certain disease states such as anxiety, depression and obsessive-compulsive disorder (OCD), can impact the cardiovagal tone and alter sympathovagal balance [4,5]. Impaired cardiovagal tone not only results in decreased HR variability and altered cardiac vagal control but also suggests emotional dysregulation, behavioural inflexibility, and lapses in executive function [6,7].

The ANS analysis evaluates neuro-cardiac function, which reflects the brain-heart interactions and dynamics of the autonomic nervous system [8]. Previous research has demonstrated a connection between decreased parasympathetic activity and conditions such as depression and anxiety disorders, as evidenced by low HR response to deep breathing (HRDB) [9-11]. Limited research has comprehensively evaluated cardiovagal tone in individuals with generalised anxiety disorder (GAD) using a full battery of autonomic function tests. Reduced cardiovagal tone observed in newly diagnosed patients with GAD has been associated with an increased risk of adverse cardiovascular outcomes in prior studies and may represent a potential physiological target for pharmacological and non-pharmacological interventions aimed at restoring neuro-cardiac balance and protective parasympathetic modulation of cardiac function.

This study was designed to systematically evaluate cardiovagal autonomic function in treatment-naïve patients with GAD using a standardised Ewing’s battery of parasympathetic function tests, including HRDB, the Valsalva manoeuvre, and the head-up tilt (HUT) test [12]. The primary aim was to compare cardiovagal tone between patients with GAD and age- and sex-matched healthy controls. In addition, exploratory analyses were undertaken to examine potential associations between cardiovagal indices and clinical variables, including severity of anxiety symptoms and sex, within the GAD cohort.

## Materials and methods

Study setting, design, and participants

This is a cross-sectional observational study conducted at a tertiary medical college between December 2023 and December 2024. It was approved by the Institutional Ethics Committee of Christian Medical College and Hospital, Ludhiana, PB, IND (approval no. BMHR-IEC/Apprvl-CMC&H/23-11-454/Psych). The study included 40 treatment-naïve patients diagnosed with GAD as per the Diagnostic and Statistical Manual of Mental Disorders (DSM)-5 criteria [13]. Healthy age and sex-matched volunteers were selected as controls.

Participants were between 31 and 40 years of age and had experienced anxiety symptoms for at least three months before their first clinical presentation. Healthy controls were clinically screened to exclude any current or past psychiatric illness, systemic medical conditions known to affect autonomic function (including hypertension, diabetes mellitus, cardiovascular disease, and endocrinopathies), substance use, or use of medications influencing the autonomic nervous system. Participants with comorbid psychiatric disorders, cardiovascular disease, diabetes mellitus, glaucoma, retinopathy, intraocular lens implantation, or use of autonomically active medications were excluded from both groups.

Cardiovascular autonomic testing was performed by trained technicians in the Autonomic Function Laboratory who were not involved in clinical assessments and were blinded to participants’ group status. To minimise potential confounding, height and weight were measured for all participants, and BMI was recorded and analysed as a continuous variable. Participants were instructed to avoid strenuous physical activity before testing and were assessed after adequate rest. Abstinence from caffeine and nicotine for at least three to four hours and alcohol for 12 hours prior to testing was ensured. During the deep breathing test, respiration was standardised at six breaths per minute in accordance with Ewing’s protocol [12]. All testing was conducted in a quiet, temperature-controlled environment following a standardised rest period.

The severity of anxiety was assessed using the Hamilton Anxiety Rating Scale (HAM-A) [14]. The diagnosis was confirmed by the consultant of the Department of Psychiatry as per the DSM-5 criteria. The 14-item HAM-A was developed by Hamilton in 1959 to detect symptoms of anxiety in clinical populations. It has since been translated into several languages in different cultures. The instrument’s robust psychometric properties include excellent reliability and construct validity in multicultural populations. The gradation of HAM-A is based on scores from 0 to 6, indicating no anxiety; 7 to 13, mild anxiety; 14 to 19, moderate anxiety; 20 to 26, high anxiety; and >26, severe anxiety [14].

Assessment of cardiovascular autonomic tone

Pre-Test Conditions

Subjects were instructed to refrain from caffeine and nicotine for at least three to four hours, and alcohol for 12 hours before testing. Testing was conducted in a quiet room with neutral temperature and humidity. The subject was allowed to rest in a supine or seated position for 30 minutes before measurement.

Autonomic Function Testing

Cardiovascular autonomic function: This was assessed using the standard protocols in the Autonomic Function Lab, Department of Neurology, at the Christian Medical College and Hospital in Ludhiana. The machines used for recording the autonomic function tests were the iVY-Cardiac Trigger Monitor 3000 (Ivy Biomedical Systems Inc., Branford, CT, USA), WR-Test Works™ Analog Interface (WR-Medical Electronics Co., Maplewood, MN, USA), BMEYE Nexfin Monitor Model 1 (Edwards Lifesciences Corporation (BMEYE), Irvine, CA, USA), and Nexfin (Edwards Lifesciences Corporation (BMEYE)).

HRDB: The subject rested for five minutes, followed by maximal deep breathing at a rate of six breaths/min (inspiratory and expiratory cycles of five seconds each). The HR response is analysed in the time domain and recorded as the mean difference between the maximum and minimum heart rates (average HRmax - HRmin). The E:I ratio assesses the ratio of the longest R-R interval during expiration to the shortest R-R interval during inspiration.

Valsalva manoeuvre: The subject blew into a mouthpiece to maintain 40 mm Hg pressure for 15 seconds. The HR was recorded before and after the manoeuvre, and the Valsalva ratio was calculated as the ratio of maximum HR to minimum HR following the manoeuvre.

HUT: The subject initially rested in a supine position for baseline recording for five minutes with straps applied across the upper chest and knees to secure the subject to the tilt table. The HUT was then performed for 10 minutes at a 70-degree angle. During the tilt, changes in HR, BP, and any symptoms experienced were continuously monitored. The 30:15 ratio is derived as the ratio between the longest RR interval around the 30th beat and the shortest RR interval around the 15th beat immediately after tilt-up.

## Results

Participant characteristics

A total of 80 participants were included, comprising 40 newly diagnosed, treatment-naïve patients with GAD and 40 age- and sex-matched healthy controls. The age range of participants was 31 to 40 years, with a mean age of 35±3.0 years. Among the GAD cohort, anxiety severity assessment using the HAM-A revealed an equal distribution, with 20 (50%) patients classified as having moderate anxiety and 20 (50%) patients classified as having severe anxiety.

There was no statistically significant difference between cases and controls with respect to height (p = 0.751), weight (p = 0.690), or BMI as a continuous variable (p = 0.641), indicating comparability in basic anthropometric characteristics. The sex distribution was identical in both groups (35% male, 65% female) and showed no statistically significant difference (χ² = 0, p = 1.00), indicating appropriate matching. The BMI category distribution among patients with GAD and age- and sex-matched healthy controls is summarised in Table 1. A statistically significant difference was observed in the distribution of BMI categories (χ² = 8.254, p = 0.041). Underweight and obese categories were more frequent among cases, whereas normal and overweight categories were more common among controls. However, BMI category distribution did not differ significantly between males and females (χ² = 5.15, p = 0.16).

**Table 1:** Distribution of BMI categories among patients with GAD and healthy controls This table presents the distribution of BMI categories among patients diagnosed with GAD and age- and sex-matched healthy controls. The BMI was classified according to the WHO criteria: underweight (<18.5 kg/m²), normal weight (18.5-24.9 kg/m²), overweight (25.0-29.9 kg/m²), and obese (≥30.0 kg/m²). Data are expressed as the number of participants (n) and percentage (%). GAD: Generalised anxiety disorder

BMI	Patients with GAD, n (%)	Healthy controls, n (%)
Underweight (<18.5)	10 (25%)	7 (17.5%)
Normal (18.5-24.9)	19 (47.5%)	24 (60%)
Overweight (25.0-29.9)	5 (12.5%)	9 (22.5%)
Obese (>30.0)	6 (15%)	0 (0%)

Results of the autonomic function tests

Autonomic function tests were performed on 40 patients, aged 31-40 years, with GAD (14 male (35%) and 26 females (65%)), who were then compared with their corresponding age- and sex-matched healthy controls. Patients demonstrated a mean HRDB of 21.61 ± 9.75 beats/min, which was significantly lower than that of the controls at 27.35 ± 9.64 beats/min (p=0.009). The E:I ratio was 1.29 ± 0.26 in patients and 1.47 ± 0.20 for controls (p=0.001). The Valsalva ratio was lower in patients (1.75 ± 0.45) than in controls (1.82 ± 0.34). However, a statistical significance could not be established (p-value=0.41). The 30:15 ratio was 0.92 ± 0.18 in patients and 0.93 ± 0.12 in controls, with a p-value of 0.726.

Normative percentiles were calculated at 2.5% and 7.5% according to age and gender. Independent sample t-tests compared the autonomic function parameters between patients with GAD and controls. The significance level was set at p<0.05. The comparative results of autonomic function tests between patients with GAD (n=40) and controls (n=40) are presented in Table 2. 

**Table 2:** Comparison of cardiovagal autonomic function parameters between patients with generalised anxiety disorder and healthy controls This table compares cardiovagal autonomic function parameters between patients with GAD and age- and sex-matched healthy controls. Parameters assessed include HRDB, the E:I ratio, Valsalva ratio, and 30:15 ratio during HUT testing. Data are presented as mean ± standard deviation. Group comparisons were performed using independent sample t-tests, and corresponding t-values and p-values are reported. A p-value < 0.05 was considered statistically significant. GAD: Generalised anxiety disorder, HRDB: Heart rate response to deep breathing, E:I: Expiratory to inspiratory, HUT: Head-up tilt

Parameter	Patients with GAD (mean±SD)	Controls (mean±SD)	p-value	t-value
HRDB (beats/min)	21.61 ± 9.75	27.35 ± 9.64	0.009	-2.648
E:I Ratio	1.29 ± 0.26	1.47 ± 0.20	0.001	-3.348
Valsalva Ratio	1.75 ± 0.45	1.82 ± 0.34	0.410	-0.829
30:15 Ratio	0.92 ± 0.18	0.93 ± 0.12	0.726	-0.352

Pearson correlation coefficients (r) and corresponding two-tailed significance values (p) between autonomic function parameters, HRDB, E:I ratio, Valsalva ratio, and 30:15 ratio, and two variables, namely the sex and severity of anxiety symptoms in patients with GAD, are detailed in Table 3. Sex was not significantly correlated with any of the assessed autonomic function parameters, including HRDB, E:I ratio, Valsalva ratio, or 30:15 ratio (all p-values > 0.05). Pearson correlation analysis was performed to assess the relationship between anxiety severity and cardio-vagal autonomic parameters within the GAD group. No statistically significant correlation was observed between anxiety severity and HRDB, E:I ratio, Valsalva ratio, and 30:15 ratio during the HUT test (all p-values more than >0.05).

**Table 3:** Pearson correlation between autonomic function parameters, sex, and severity of anxiety symptoms in patients with GAD This table shows the Pearson correlation coefficients (r) and corresponding two-tailed significance values (p) between autonomic function parameters, i.e., HRDB, E:I ratio, Valsalva ratio, and 30:15 ratio, and two variables, namely sex and severity of anxiety symptoms in patients with GAD. Pearson correlation analysis was used, and statistical significance was set at p < 0.05. GAD: Generalised anxiety disorder, HRDB: Heart rate response to deep breathing, E:I: Expiratory to inspiratory

Parameter	Statistical measure	HRDB	E:I ratio	Valsalva ratio	30:15 ratio
Sex	Pearson correlation	0.007	0.094	-0.094	0.191
	p-value	0.966	0.565	0.564	0.237
Severity of anxiety symptoms	Pearson correlation	-0.067	0.038	0.193	0.13
	p-value	0.683	0.814	0.234	0.425

## Discussion

Most prior studies measuring dysautonomia in anxiety disorders have relied on isolated HR variability measures, which offer a limited snapshot of autonomic regulation. In contrast, we used Ewing’s battery for a standardised, non-invasive, sensitive, and reproducible set of autonomic function tests widely regarded as the clinical gold standard for assessing cardiovagal tone [15]. We recorded HR variability responses to deep breathing, the Valsalva manoeuvre, and the HUT test to assess cardiovagal tone in treatment-naïve patients with newly diagnosed GAD. This approach deliberately provoked multiple reflex components of cardiovagal control, including respiratory sinus arrhythmia and baroreflex-mediated heart rate modulation, allowing a more specific and comprehensive evaluation than single HR variability measurements alone [12]. 

Our study found HRDB to be statistically significant, suggesting an alteration in cardiovagal tone, which is considered a marker of cardiovascular health, cardiac adaptability, cognitive function, and behavioural flexibility. Lowered cardiovagal tone signals withdrawal of protective cholinergic influence on the heart and suggests alteration in the cardiac-neural axis, increasing the chances of cardiovascular complications in the future [16]. A study conducted by Jha et al. showed that the HR response to deep breathing is statistically insignificant [17]. A similar study conducted with depressed patients with a secondary diagnosis of GAD had the largest decrease in HR variability [18]. 

We also found that the E: I ratio (measured during deep slow breathing) also emerged as a particularly sensitive indicator of autonomic changes. This finding is consistent with observations made by Loukrakpam et al. [19]. The significant difference in the E:I ratio between cases and controls (p < 0.05), coupled with the quantitatively lower value of 30:15, indicates decreased parasympathetic function (lower cardiac vagal tone). This finding is similar to the studies by Friedman et al. [20] and Hoehn-Saric et al. [21]. 

Anxiety disorder patients frequently exhibit autonomic rigidity, meaning their physiological responses lack normal variability and flexibility. This reduced autonomic adaptability, particularly diminished HR variability, has been closely linked to heightened risks of serious cardiovascular conditions such as coronary heart disease, myocardial infarction, and cardiac arrhythmias, all of which can be life-threatening [22]. Previous studies have suggested an association between the severity of anxiety symptoms and dysautonomia. However, in our study, despite an equal distribution of moderate and severe anxiety cases, cardiovagal indices did not demonstrate a linear relationship with symptom severity [23].

Over the past century and a half, the brain-heart connection, initially articulated by Claude Bernard, has become a focal point in understanding how physiological and psychological processes intertwine [24]. Central to this interface is the vagus nerve, which orchestrates the integration of cardiac function, behaviour, and cognition [25]. Two influential frameworks, the 'polyvagal theory 'and the 'neurovisceral integration model', provide complementary lenses through which to view these complex relationships [26,27]. According to the neurovisceral integration model, key neural structures, including the ventromedial prefrontal cortex, anterior cingulate, and insula, form part of the central autonomic network (CAN) [28]. These overlapping circuits regulate emotional expression, executive control, behavioural adaptability, and cardiovagal tone, illustrating a shared neuroanatomical substrate for cognitive and emotional self-regulation as well as autonomic flexibility. Taken together, these theoretical and empirical advances underscore how diminished vagal-mediated HR variability is linked to impaired emotional regulation, attentional control, and behavioural rigidity mechanisms implicated in both psychological distress and physical health outcomes [29,30]. 

Given the cross-sectional design of this study, no direct conclusions can be drawn regarding future cardiovascular outcomes in patients with GAD. Rather, the observed reduction in respiratory-mediated cardiovagal indices may represent an early physiological alteration in autonomic regulation that warrants further longitudinal investigation. Importantly, parasympathetic impairment was not uniform across all cardiovagal domains. While HRDB and the E:I ratio were significantly reduced in patients with GAD, the Valsalva ratio and 30:15 ratio during HUT testing did not differ significantly from controls. These findings suggest a selective attenuation of respiratory sinus arrhythmia-mediated vagal control, rather than a generalised failure of parasympathetic reflexes. The preservation of Valsalva and orthostatic cardiovagal responses may reflect differential sensitivity of autonomic reflex pathways or indicate that certain components of cardiovagal regulation remain intact in the early stages of anxiety disorders. This pattern supports the concept of domain-specific autonomic vulnerability, rather than global parasympathetic dysfunction. Longitudinal studies incorporating cardiovascular endpoints are required to determine whether these early cardiovagal alterations translate into clinically meaningful cardiovascular risk and to evaluate the potential reversibility of these changes with therapeutic interventions.

Limitations

The relatively small sample size may limit the statistical power and generalisability of the findings. Although efforts were made to ensure homogeneity by excluding individuals on medications and those with conditions contraindicated for autonomic testing (such as glaucoma, retinopathy, and raised intracranial pressure), the age range (31 to 40 years) may have introduced age-related variability in autonomic responses. As a cross-sectional study conducted at a single centre, causal inferences cannot be drawn, and the findings may not be generalisable to broader or more diverse populations. Furthermore, the absence of longitudinal follow-up prevents assessment of changes in autonomic function with treatment or disease progression.

## Conclusions

Treatment-naïve patients with GAD demonstrate selective alterations in cardiovagal function, particularly in respiratory-mediated parasympathetic indices, suggesting early disturbances in autonomic regulation. These findings provide preliminary evidence of altered neuro-cardiac interactions in GAD; however, given the cross-sectional design, the results should be interpreted as associative and hypothesis-generating rather than causal or predictive. Future research should include longitudinal studies to determine the temporal stability and clinical significance of cardiovagal alterations and to evaluate their potential role as prognostic indicators for disease progression, treatment response, and cardiovascular outcomes. Interventional studies examining pharmacological, behavioural, and lifestyle-based treatments are also needed to assess whether improvement in anxiety symptoms is accompanied by restoration of autonomic balance. Such investigations will help clarify the clinical utility of standardised cardiovagal assessment and determine whether autonomic function testing can be meaningfully incorporated into routine evaluation and monitoring of patients with anxiety disorders.
